# Titanium mesh and pedicled buccal fat pad for the reconstruction of maxillary defect: case report

**DOI:** 10.1186/s40902-021-00295-6

**Published:** 2021-03-17

**Authors:** Joo-Hyung Yoon, Young-Wook Park, Seong-Gon Kim

**Affiliations:** Department of Oral and Maxillofacial Surgery, College of Dentistry, Gangneung-Wonju National University, Jukheon gil 7, Gangneung, Gangwondo 25457 Republic of Korea

**Keywords:** Maxilla, Reconstructive surgical procedures, Surgical mesh, Adipose tissue

## Abstract

**Background:**

Pedicled buccal fat pad (PBFP) has been used for the reconstruction of small-sized maxillary defects but cannot be used without hard tissue support on the defect larger than 4 cm × 4 cm × 3 cm.

**Case presentation:**

A 64-year-old man had a history of squamous cell carcinoma of the left maxilla. After removal of the posterior maxilla, a complex bone defect (size, 5 cm × 4 cm × 3 cm) was immediately reconstructed using PBFP combined with a titanium mesh. A pinpoint fistula was found in the left palatal region 1 month after the surgery and was treated with a palatal sliding flap. There were no further complications during the follow-up.

**Conclusion:**

The present technique demonstrated that PBFP combined with a titanium mesh could be used for the reconstruction of complex maxillary defect (size, 5 cm × 4 cm × 3 cm) without additional bone graft.

## Background

Pedicled buccal fat pad graft (PBFP) is one of the procedures used for the reconstruction of maxillary defects such as oroantral fistula, cleft palate, medication-related jaw bone necrosis, and defects formed after cysts or tumors removal [1]. It is widely used because it has advantages such as rich vascularity, proximity to the recipient site, technical simplicity, and high success rate [1, 2]. Other methods for the reconstruction of maxillary defects are free skin graft, buccal advancement flap, palatal pedicled flap, and microvascular flap [3, 4].

The reconstruction method is selected based on the size of the defect and the anatomical location. In the case of small-sized defects, direct closure or PBFP can be considered [1, 5]. For the reconstruction of a large maxillary defect, a free vascularized graft or pedicled flap should be used for closure [6]. It is difficult to perform PBFP for large oral defects because of the limitations in size. When there is a sound supporting structure, many authors recommend reconstructive surgery with PBFP for defects smaller than 4 cm × 5 cm [2, 7, 8]. Rapidis et al. [9] mentioned that the failure rate of PBFP is high when performed on defects larger than 4 cm × 4 cm × 3 cm. If there is no supporting hard tissue in the defect, PBFP should be used only for small defects such as oroantral fistula after tooth extraction [5]. If PBFP is used for the coverage of larger defects, the additional bone graft is essential as supporting structure [1].

After maxillary tumor resection, the supporting structures are removed. Therefore, solitary PBFP cannot be used for this type of defect. In this case, a titanium mesh was used as the supporting structure for PBFP. In this study, maxillary defect (size, 5cm × 4cm × 3cm) was successfully reconstructed using a PBFP combined with a titanium mesh without bone graft.

## Case presentation

**Fig. 1:**
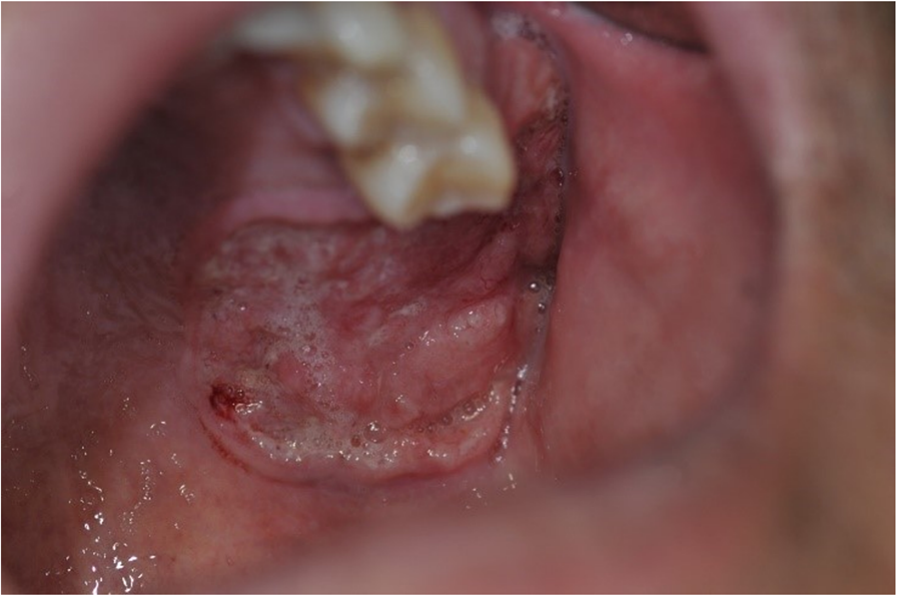
Pre-operative intra-oral finding. The ulcerated lesion was shown on the left posterior maxillary edentulous region

**Fig. 2:**
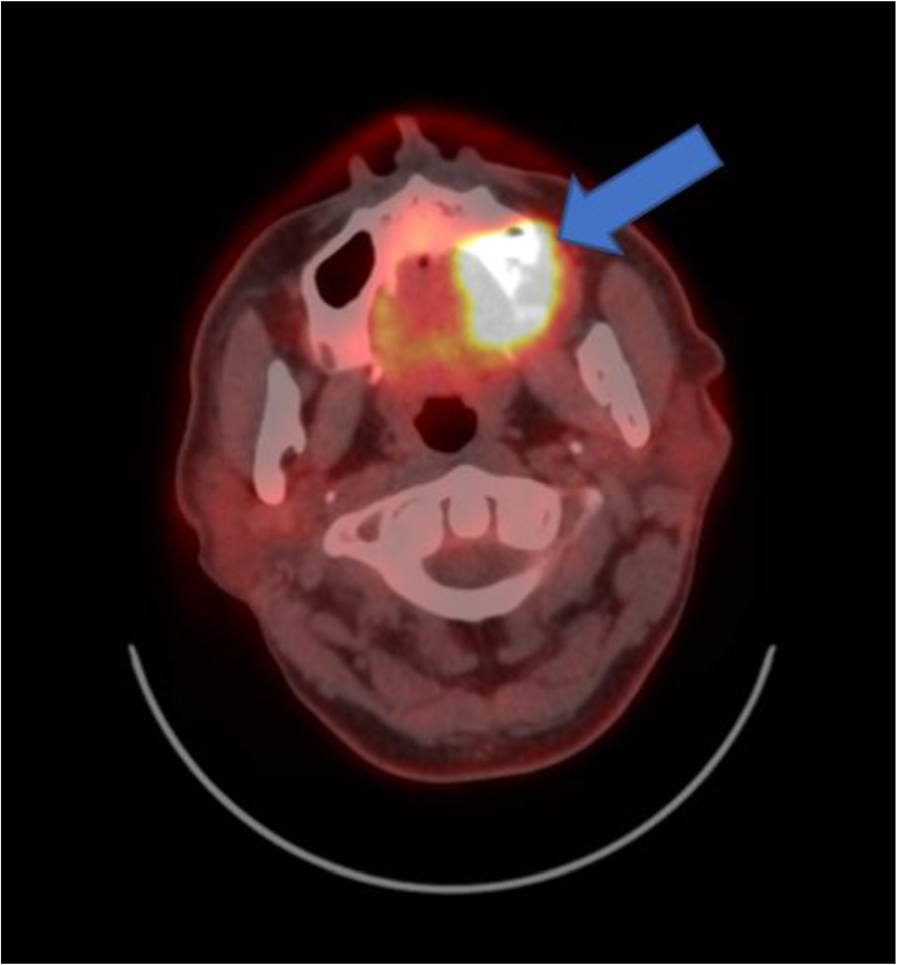
PET-CT image. The lesion was shown on the sinus floor of the left maxilla (arrow)

**Fig. 3:**
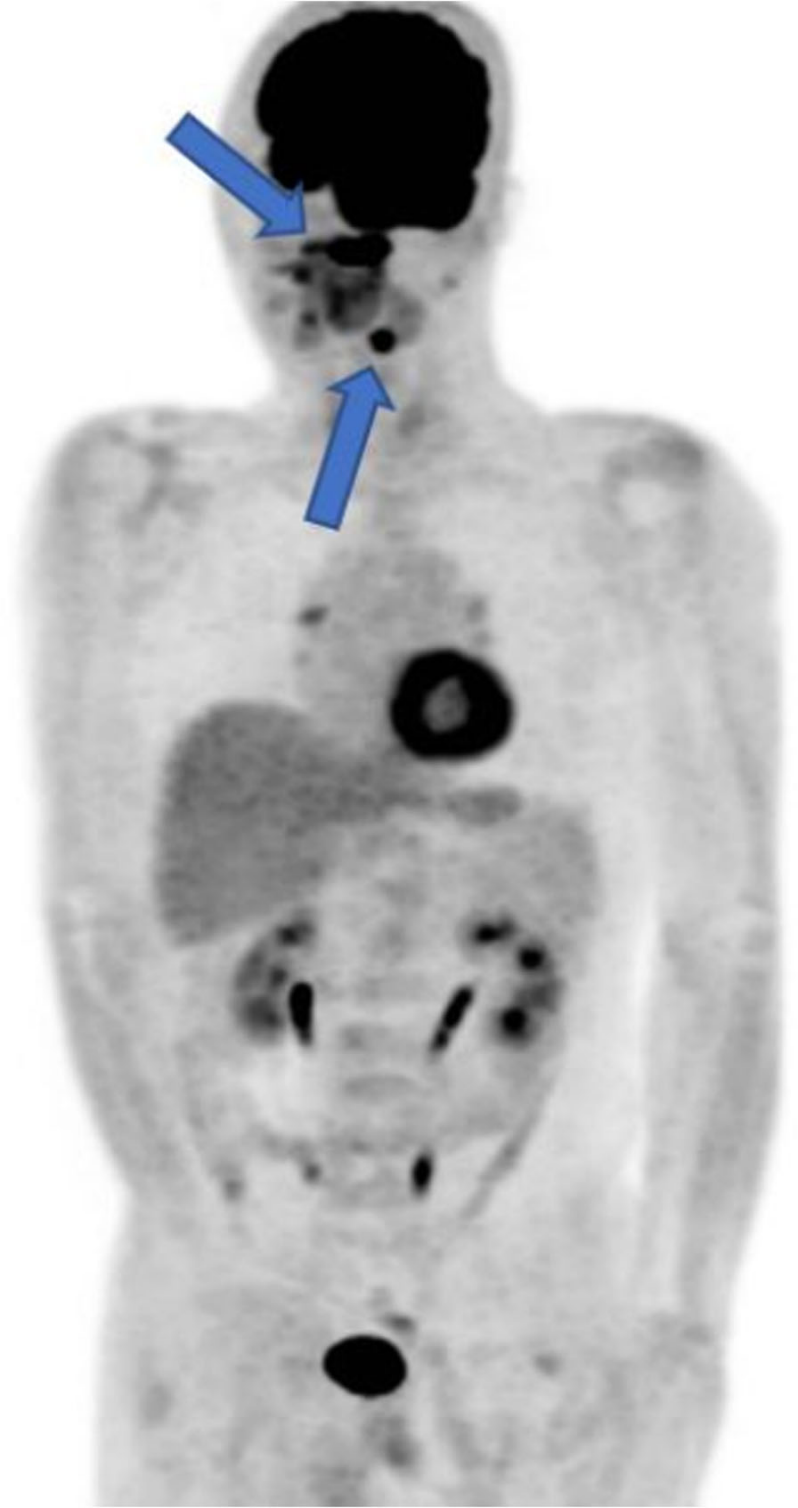
Torso PET-CT image. The lesion was located on the left maxilla. Regional lymph node metastasis was found on the left cervical level IB and II

A patient complained of pain in the left posterior edentulous region of the maxilla for 1 year. The pain was observed after the extraction of the left maxillary molars. During the clinical examination, a 3 cm-sized ulcerated lesion was observed in the left posterior edentulous region (Fig. 1). A biopsy was performed, and according to the result of the pathological examination, the lesion was diagnosed to be a well-differentiated squamous cell carcinoma. PET-CT was performed and the result showed that the locations were the alveolar process and the sinus floor of the left maxilla. In addition, regional lymph node metastases were found in the left cervical IB and left cervical II (Figs. 2 and 3). Accordingly, the TNM stage was T2N2bM0 (stage IV).

**Fig. 4:**
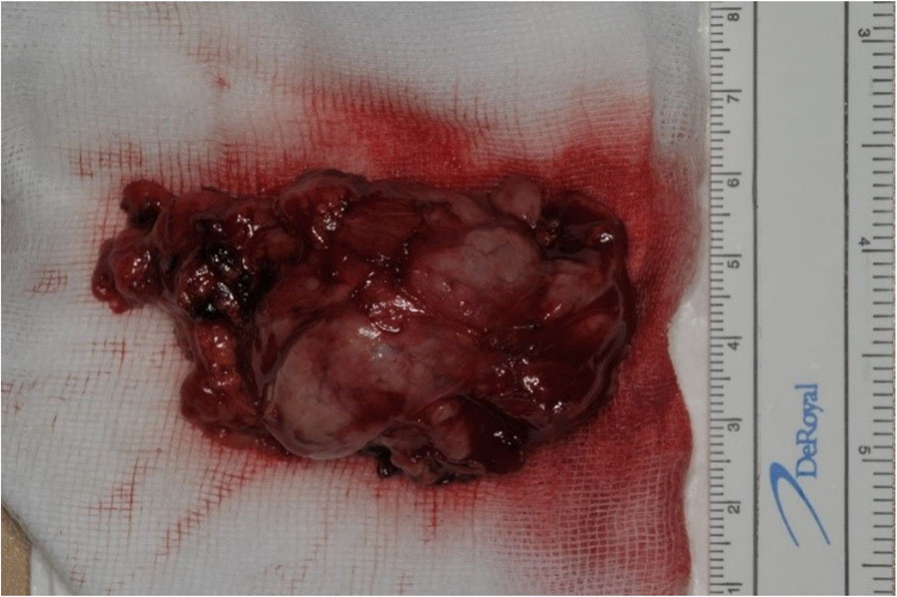
Main mass after partial maxillectomy

On August 6, 2020, selective neck dissection and partial maxillectomy were performed under general anesthesia (Fig. 4). As the patient had a stage IV malignant tumor, immediate reconstruction using regional tissue was considered. Complex maxillary defect exposed the maxillary sinus. The lateral, posterior, and inferior walls of the sinus were partially removed. The size of the bony defect in the maxilla was measured at post-operative CT scan and it was 50 mm × 36 mm × 28 mm (length × width × height).

**Fig. 5:**
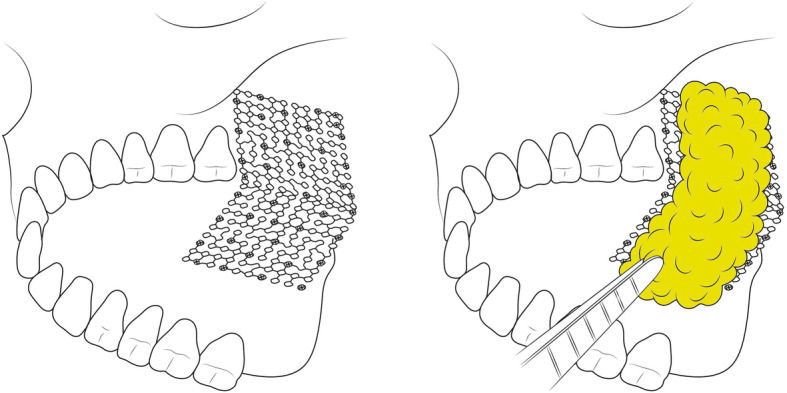
Illustration for surgical technique. Mesh was applied on the maxillary defect and fixed on the remaining maxilla with screws. Then, the mesh was covered with pedicled buccal fat pad graft. Pedicled buccal fat pad graft was fixed to resection margin with 4-0 vicryl

For the closure of the defect between the oral cavity and the sinus, PBFP flap and mesh application was performed. The mesh was used to determine the role of the bone, which was the base of the graft. The mesh was adjusted and fixed on the remaining maxilla using screws (Fig. 5). The mesh was covered by a flap, and the flap was sutured with the mesh and the adjacent soft tissues. The stent was applied to protect the surgical site.

**Fig. 6:**
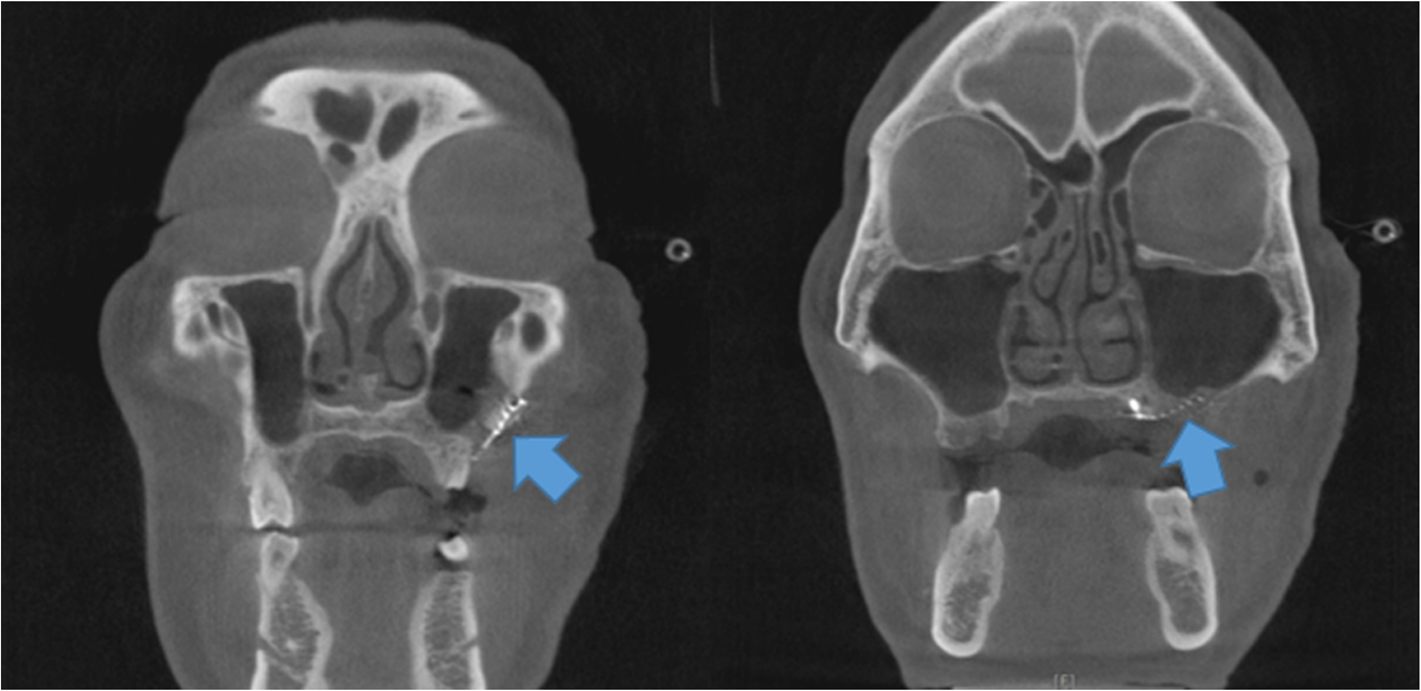
Post-operative CT scan. Maxillary bony wall defect was reconstructed with titanium mesh (arrows). The affected maxillary sinus (left) was healthy and there was no mucosal thickening

**Fig. 7:**
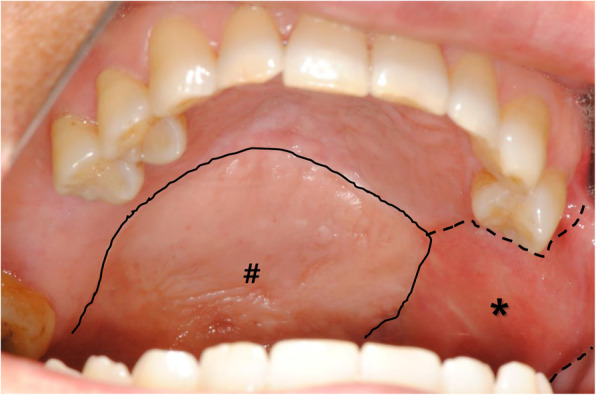
Six months after pedicled buccal fat pad graft surgery and 5 months after palatal rotation flap surgery. Rotated palatal flap (#) showed similar color to adjacent palatal mucosa. The area covered with pedicled buccal fat pad graft (*) showed reddish compared to adjacent oral mucosa. The surgical site was healed by the oral mucosa and color was matched to adjacent tissues

There was no infection or disruption to the surgical site until 1-month post-surgery (September 4, 2020). However, on September 8, 2020, the patient complained of leakage of fluid into the nasal cavity and one pinpoint fistula was found (1.5 mm in size) in the border between the regenerated mucosa and resection margin. It was located in the distal region of the left first premolar area. For the closure of the fistula, a palatal rotation flap was done. Post-operative CT scan demonstrated that there was no evident inflammation in the left maxillary sinus, and sinus walls were successfully repaired by titanium mesh (Fig. 6). Until 6 months postoperatively (February 8, 2021), no further fistula was observed, and the wound healed without any complications (Fig. 7). Adjuvant therapies for tumor such as chemotherapy or radiotherapy were not performed after the surgeries.

## Discussion

For the reconstruction of large maxillary defects, the parieto-temporal flap [10] or micro-vascularized free flap harvested from the scapula [6], fibula [11], or forearm [12] is used. The advantages of these flaps are the simultaneous reconstruction of soft and hard tissues and are applicable even in poor recipient beds. In addition, these flaps are too bulky to reconstruct the palatal area. Some flaps can reconstruct both soft and hard tissues [6, 11]. Donor site morbidity, long operation time, and expensive treatment fees are the disadvantages of these techniques [1]. Compared to these techniques, PBFP combined with titanium mesh is a simple and inexpensive technique. In this case presentation, large defect after removal of squamous cell carcinoma was successfully reconstructed using PBFP combined with titanium mesh.

PBFP has been widely used for the reconstruction of oral defects because it is simple, the graft has rich vascularity, and the technique has a high success rate [1, 2]. However, PBFP is a fragile soft tissue flap that is easily torn during flap harvesting. Therefore, PBFP cannot be applied to defects without supporting structures. Maxillary sinus wall is frequently removed during tumor resection. If the bony wall is removed, the PBFP-only flap cannot prevent fluid and air leakage.

In this case, a titanium mesh was used as the supporting structure for PBFP. The defect and mesh were covered by a PBFP flap. Even though the defect was large (50 mm × 36 mm × 28 mm), the reconstruction was successful. Almost all parts of the defect were perfectly epithelialized after surgery, except for the occurrence of a pinpoint fistula. The fistula was treated using a palatal rotation flap. Regardless of fistula size, palatal fistula is hardly closed by direct closure. Accordingly, residual palatal mucosa was transposed laterally. As shown in postoperative follow-up image (Fig. 7), most surgical defect was covered with transformed mucosa from buccal fat tissue.

In the case of maxillary anterior alveolar bone defect, custom-made titanium mesh combined with bone graft is a good option for reconstruction [4]. However, custom-made titanium mesh combined with bone grafts can be performed in cases with sufficient soft tissue coverage. Therefore, this technique was inappropriate in our case. This case presentation showed that complex maxillary defects without hard tissue on the base could be reconstructed with PBFP combined with titanium mesh. Maxillary defect due to cancer treatment has been treated by PBFB in previous publication [13]. In previous technique [13], PBFB was used with titanium mesh for the reconstruction of sinus mucosa. Oral mucosa was covered with regional mucosa flap [13]. Subsequent technique from the same team added bone graft to this technique, but PBFB was still used for sinus repair [14]. This technique cannot be used for large oral mucosal defect where it cannot be covered with regional mucosal flap. As titanium mesh has pore, stem cells in the adipose tissue may migrate to both oral and sinus area. In postoperative CT scan, the titanium surface of sinus area was also covered with regenerated soft tissue (Fig. 6).

As patient did not receive either radiation therapy or chemotherapy, the risk of mesh exposure was not clarified in this study. Radiation is a risk factor for the implant loss [15]. The radiation-induced failure is still high for the implant in vascularized bone flap [16]. In case of custom-made titanium mesh, titanium mesh exposure is observed after radiation therapy [17]. Compared to other types of flap, PBFB might not be more protective to radiation therapy or chemotherapy. Considering that there is no flap to protect implant from the radiation therapy or chemotherapy, perfectly, any reconstruction should be delayed until finishing radiation therapy. If there was any reason to reconstruct surgical defect before additional tumor therapy, PBFB combined titanium mesh could be a candidate method because of its simplicity and minimal donor site morbidity.

## Conclusions

This case presents a new solution for the reconstruction of a large maxillary defect without a supporting structure, which many authors previously recommend microvascular graft or pedicled muscle flap for reconstruction. The coverage of the PBFP flap on large maxillary defects has more advantages compared to previous methods, such as low donor site morbidity, technical simplicity, reduced operation time, and low cost.

## Data Availability

Data sharing is not applicable to this article since no dataset was generated or analyzed during the current study.

## Abbreviation

PBFP: Pedicled buccal fat pad
